# Spatial-temporal dynamics and driving factors of urban construction land in mega cross-river mountain cities: A case study of Chongqing’s central urban area, China

**DOI:** 10.1371/journal.pone.0315943

**Published:** 2025-01-16

**Authors:** Fu-hai Wang, Wei Zeng, Dan Chen, Chang-hua He, Hui Li

**Affiliations:** 1 College of Architecture and Urban Planning, Chongqing University, Chongqing, China; 2 ChongQing Finance and Economice College, Chongqing, China; East China Normal University, CHINA

## Abstract

The evolutionary model of construction land serves as a fundamental pillar in national spatial development and planning research. However, previous studies have overlooked the "climbing" mode of construction land on three-dimensional terrains. To address this issue, utilizing elevation data and land use data from 2010 to 2020, this study employs slope analysis, intensity analysis, spatio-temporal transformation, and PLUS model to elucidate the spatial expansion process and driving forces of urban construction land in Chongqing from both two-dimensional and three-dimensional perspectives. The findings indicate that: (1) From a three-dimensional topographical standpoint, between 2010 and 2012, construction land gradually expanded towards low-slope areas, whereas between 2012 and 2020, it progressively extended into high-slope regions. (2) Regarding land type conversion patterns, the shift from arable land to construction land demonstrates a systematic inclination, while other transformations exhibit absolute or relative tendencies. Conversely, the conversion from construction land to arable land also displays a systematic pattern. (3) Since 2010, the growth process of construction land has transitioned from slow-equilibrium to rapid-disequilibrium with an expanding spatial disparity. (4) Most areas maintain relatively stable spatial conditions without significant jumps or transitions observed. (5) The expansion of construction land in Chongqing is primarily influenced by terrain, river, tunnel, rail transit, and other factors. The outcomes of this study can provide scientific foundations and decision-making references for rational planning in similar cities characterized by mountainous landscapes intersected by rivers.

## 1 Introduction

Construction land plays a crucial role in ensuring industrialization, urbanization, and economic and social development, and its expansion represents the fastest and irreversible process of human-induced land use change [1, 2]. Since the 1980s, regional development strategies such as Western Development Program and Chengdu-Chongqing Economic Circle have accelerated the urbanization process of cities in western China. The expansion of urban construction land serves as a direct reflection of urbanization and land use change. However, these cities were divided across mountains and rivers. Their complex terrain, special geology, and limited flat areas pose challenges for spatial expansion [3, 4], while simultaneously fostering a unique form of inter-mountain urban development [5–7]. Therefore, it is highly significant to explore its temporal and spatial evolution characteristics along with Driving factors within the context of new-type urbanization. This exploration will aid in establishing an effective mechanism for coordinated regional development [8–10].

Currently, the spatial expansion of construction land has emerged as a prominent research topic, with numerous scholars investigating the patterns and Driving factors underlying this phenomenon.

Regarding the expansion mode of urban construction land, the current research focuses on the type of expansion mode and its spatio-temporal pattern. The earliest model is the concentric circle model, followed by the fan expansion model and multi-core growth model [11, 12]. Furthermore, in response to the spatial development pressure caused by urban expansion, the theory of compact cities was introduced, which emphasizes minimizing non-urban land use during urban expansion [13–16]. Building upon these models, researchers have analyzed the spatio-temporal patterns of urban construction land expansion [17–19]. For instance, the China Land Use Database (CLUD) has been utilized to examine the trajectory and spatio-temporal patterns of urban land expansion in China [20, 21]. Various indicators such as average annual expansion area, type of urban expansion, and fractal dimension have been employed to analyze characteristics and regional differences in China’s 40-year period of urban expansion as well as its impact on land use and urban spatial form [22]. Current research mainly focuses on the "two-dimensional" expansion of plain cities, using traditional research methods such as construction land expansion intensity and transfer matrix, while neglecting the "three-dimensional" expansion of mountainous cities in western China. The construction land of mountainous cities should not only consider horizontal expansion, but also vertical expansion, that is, "three-dimensional" expansion [23, 24].

In the term of Driving factors for urban construction land expansion, existing research differences primarily lie in the driving factors and study areas [25]. The current studies predominantly focus on population, economy, topography, policy, and technology to examine their impact on urban construction land [26]. For instance, cities situated across rivers have been explored to understand the process and dynamic mechanism of urban spatial growth [4, 27–29]. Similarly, extensive research has been conducted on the influence of bridges and roads on the expansion of urban construction land [30–32]. However, existing research in China is mainly concentrated in the eastern coastal regions. Notably, studies have extensively investigated the evolution and driving forces behind urban land spatial patterns in eastern city clusters such as Beijing-Tianjin-Hebei [8, 33–38] and Guangdong-Hong Kong-Macao Greater Bay Area [39]. While a significant number of researchers have examined the Driving factors for urban expansion in these eastern coastal areas, further investigation is required to determine if these mechanisms can be applied to the western mountainous regions of China [40, 41].

Considering the aforementioned challenges, this study focuses on investigating the spatio-temporal characteristics and Driving factors of urban construction land across mountains and rivers in China. The key contributions of this research are as follows: (1) By utilizing large-scale and high-resolution data, we provide insights into the three-dimensional development model of urban construction land in China. (2) Through a case study conducted in Chongqing, a central city in western China, we analyze and summarize the Driving factors behind urban construction land expansion in mountainous areas.

The remaining parts of this paper are organized as follows. Section 2 describes the study area. Section 3 introduces a comprehensive description of data sources and research methodology. Section 4 analyzes empirical results. Finally, Section 5 concludes with key findings and discusses potential avenues for future research.

## 2 Overview of the study area

**Fig 1:**
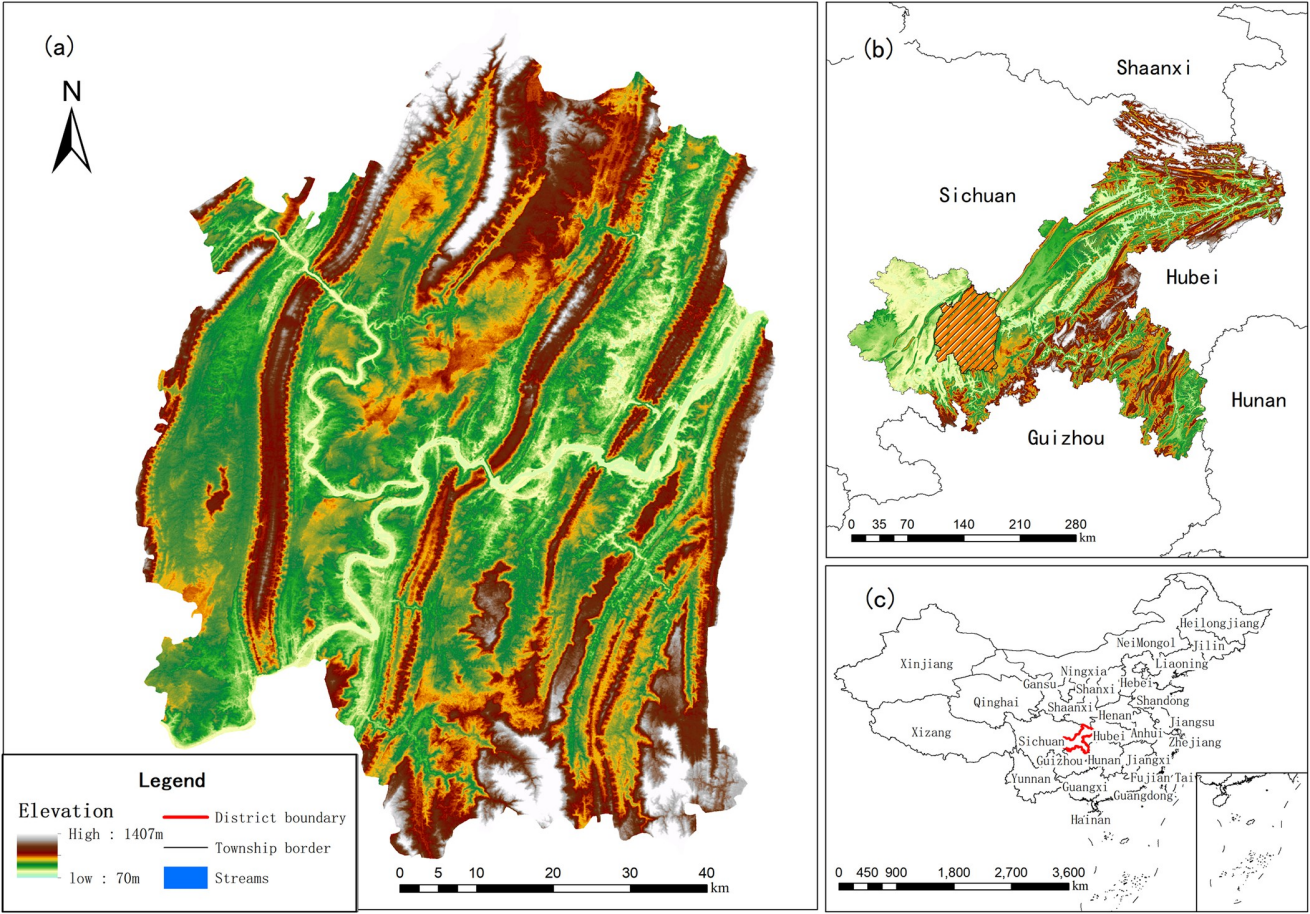
Location map of Chongqing central urban area. Note:DEM data is sourced from the Geospatial Data Cloud website (http://www.gscloud.cn/home).Administrative boundary data comes from Resource and Environment Science and Data Center (https://www.resdc.cn/), and the map boundary has not been changed. Cartographic software:ArcGIS.

This study focuses on the central urban area of Chongqing, China, with a total area of approximately 5,465 square kilometers, accounting for approximately 6.63% of the total area of Chongqing municipality. As of 2020, the built-up area covers approximately 1,089 square kilometers, constituting approximately 19.92% of the central urban area of Chongqing. With elevations ranging from 168 to 693 meters and a height difference of nearly 500 meters, the confluence of the Jialing River with the Yangtze River at Chaotianmen forms the Yuzhong Peninsula, characterizing it as a typical large cross-river mountainous city, As shown in Fig 1. The central urban area of Chongqing serves as a key center for economic development within the Chengdu-Chongqing Economic Circle, playing a crucial role in driving rapid economic growth in the southwestern region of China. Choosing the central urban area of Chongqing as the study subject is not only representative in terms of importance, uniqueness, and typicality but also holds both theoretical and practical significance for researching the spatial growth of large cross-river mountainous cities.

## 3 Materials and methods

### 3.1 Data sources

**Fig 2:**
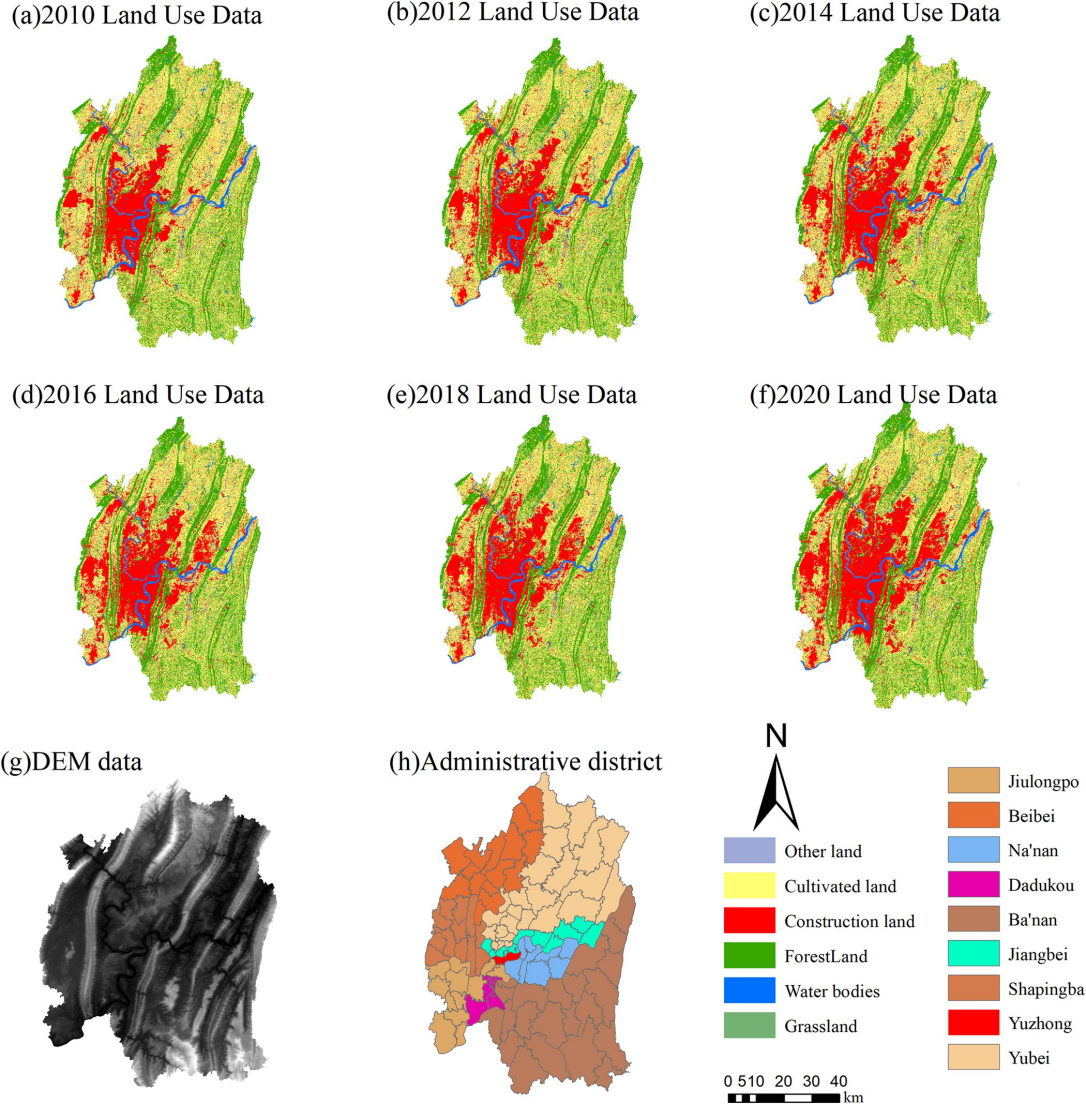
Land use data, DEM data, and administrative district data in the central urban area of Chongqing. Note: The basemap was obtained from the Geospatial Data Cloud(http://www.gscloud.cn/home), and the map boundary has not been changed.Cartographic software:ArcGIS.

The data utilized in this research primarily consists of three components: (1) The annual land change survey database of the central urban area of Chongqing for various years (2010, 2012, 2014, 2016, 2018, and 2020), with a scale of 1:10000. (2) Township level administrative boundary data, sourced from the integration of resource and environmental science data (https://www.resdc.cn/) There are a total of 114 townships, as illustrated in Fig 2. (3) Data on driving factors of land use change, including population, GDP, soil types, rivers, annual average rainfall, transportation, government locations, water body and annual average temperature, sourced from the Resource and Environmental Science and Data Center website (https://www.resdc.cn/). Elevation data are obtained from the Geographic Spatial Data Cloud website (http://www.gscloud.cn/home), as outlined in Table 1.

**Table 1:** Spatial driving factors of urban construction land change in this study.

Category	Data type	Year	Original resolution	Data source
Social and economic factors	Population	2020	1km	Resource and Environmental Science and Data Center website (https://www.resdc.cn/)
	GDP	2020	1km	
	Distance to urban expressways	2020	30m	Chinese Geographic Information Resource Catalog Service System, which uses Euclidean distance for calculations
	Distance to main roads	2020	30m	
	Distance to secondary roads	2020	30m	
	Distance to district government offices	2020	30m	
	Distance to tunnels	2020	30m	
	Distance to bridges	2020	30m	
	Distance to railways	2020	30m	
	Distance to subway stations	2020	30m	
Climate and environmental factors	Distance to major rivers	2020	30m	Chinese Geographic Information Resource Catalog Service System, which uses Euclidean distance for calculations
	Annual average temperature	2020	1km	Resource and Environmental Science and Data Center website (https://www.resdc.cn/)
	Annual average precipitation	2020	1km	
	Soil types	2000	1km	
	Elevation data	2019	30m	Geographic Spatial Data Cloud
	Slope data	2019	30m	Generated from elevation data

### 3.2 Research procedure

**Fig 3:**
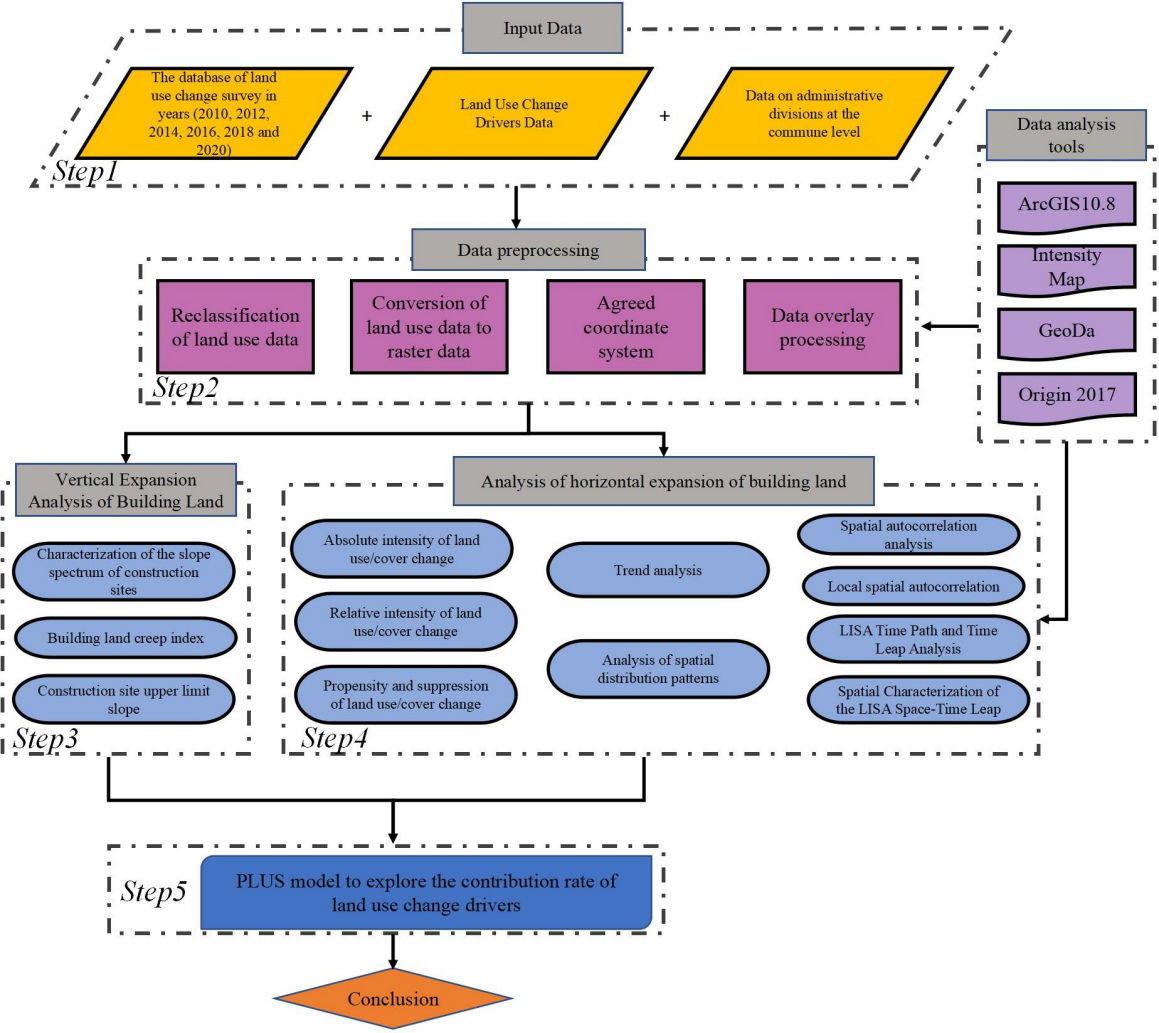
Flowchart for characterizing the spatial and temporal dynamics of urban built-up land.

The research framework consists of 5 steps, as illustrated in Fig 3:

1. To ensure the accuracy of the study, high-precision, and high-temporal-resolution land-use data, land-use change driver data, and administrative district data are employed.
2. Since the driver data originates from multiple sources, it is essential to standardize the data to the WGS 84 coordinate system for ease of spatial operations. Analytical and processing tasks are then carried out using software such as ArcGIS 10.8, GeoDa, Origin 2017, and Intensity Map. Furthermore, to simplify calculations and statistical analysis, the current land-use data is converted to an "unsigned char" format and reclassified into six major categories: cultivated land, forest land, grassland, construction land, water bodies, and other land. All raster data is resampled to a 30 m × 30 m grid, as indicated in Table 2.
3. Calculate the slope spectrum characteristics of construction land, the construction land climbing index, and the upper limit slope to analyze the vertical expansion of construction land in the study area based on these results.
4. Analyze the LUCC (Land Use and Land Cover Change) intensity spectrum, spatial distribution patterns, global spatial autocorrelation, LISA (Local Indicators of Spatial Association) temporal pathways, and spatiotemporal transition analysis in the study area to observe the characteristics of horizontal expansion of construction land.
5. Utilizing the PLUS model, initially employ the "Extract Land Expansion" module to analyze land expansion from 2010 to 2020. Subsequently, LEAS (Land Expansion Analysis System) will be employed to explore the driving factors behind construction land expansion.

**Table 2:** Reclassification of land use status data.

Reclassify	Cultivated land	Woodland	Grassland	Construction land	Waters	Other land
Reclassification encoding	1	2	3	4	5	6

### 3.3 Methods

#### 3.3.1 Vertical expansion of built-up

1. *Construction of the slope spectrum of construction land*. To quantitatively and thoroughly explore the evolution characteristics of construction land in terms of slope in the central urban area of Chongqing, this study relies on slope data and land-use data for six different periods. It involves calculating and tallying the number of grid cells within various slope ranges at 1° intervals within the region and the number of grid cells designated as construction land. This data is then used to generate topographic slope spectra and construction land slope spectra. The concept of slope spectra is derived from the geoscientific analysis method that Zhang Hanxuan proposed when studying the topography and geomorphology of the Lanzhou-Xining urban cluster in China [39]. The concept of slope spectrum curves originated from the research on the topography and landforms of the Lanzhou-Xining urban agglomeration conducted by Zhang Hanxuan and others in China. The slope spectrum calculated in this study encompasses the topographic slope spectrum (i.e., the frequency-area slope spectrum of natural land) and the land use slope spectrum (i.e., the frequency-area slope spectrum of developed land) [39]. The specific calculation formulas are as follows:
$$P_{t,i}=\frac{A_{t,i}}{A_{t}}\times 100\%$$ (1)
$$P_{cl,i}=\frac{A_{cl,i}}{A_{cl}}\times 100\%$$ (2)
  In the formula, At is the total land area in the study area, *A*_*cl*_ is the total area of construction land in the study area, *A*_*t*,*i*_ is the area of land that is the slope of *i*, *A*_*cl*,*i*_ is the area of construction land with slope of *i*, *P*_*t*,*i*_ is the area frequency (%) of land in the area with slope of *i*, and *P*_*cl*,*i*_ is the area frequency (%) of construction land with slope of *i*. In the slope spectrum graph, the x-axis represents the slope frequency, and the y-axis represents the area frequency. When the terrain slope spectrum curve intersects with the construction land slope spectrum curve, the slope value at that point is called the *T*-value (°). Using the T-value (°) as a turning point, the intensity of the construction land climbing can be analyzed.
2. *Construction land creep index and upper limit slope*. Visual interpretation of the slope spectrum of built-up land can characterize the slope gradient and spatial and temporal changes in the distribution of built-up land. However, there is still a need to analyze the specific degree of creep on the construction site on the basis of quantitative indices. To this end, this paper defines the Built-up Land Climbing Index (BCI) by calculating the change in the proportion of the area of built-up land in areas with slopes higher than T to the total area of built-up land over a period, which is calculated by the following formula:
$$BCI=(\frac{l_{j}}{L_{j}}-\frac{l_{i}}{L_{i}})\times 100\%$$ (3)
  Where *l*_*i*_ is the area of construction land with a slope above T at time point *i*, *l*_*j*_ is the area of construction land with a slope above T at time point *j*, *L*_*i*_ is the total area of construction land at time point *i*, and *L*_*j*_ is the total area of construction land at time point *j*. The *BCI* is the upper limit slope change value (*ULSC*). When *BCI*<0, it indicates that there is no slope-climbing phenomenon, and when *BCI*>0, it indicates that there is a slope-climbing phenomenon. The value of change in upper limit slope (*ULSC*) is used to determine whether urban construction is moving towards higher slopes. When *ULSC*>0, it indicates that the upper limit slope of the urban construction land increases and moves towards higher slope areas, and the larger the value, the greater the magnitude of the slope climbing [39].

#### 3.3.2 Strength analysis framework

The absolute intensity of land use/cover change reflects the absolute amount of transformation between land classes in a certain time interval, which can be analyzed from two perspectives: transferring from one land class to another and from other land classes to this land class. Based on the relative intensity of land use/cover change, the influence of the conversion intensity between land classes on the land use/cover structure of the study area was further analyzed [42, 43].

1. *Absolute intensity of land use/cover change*. Eq (4) calculates the absolute transfer intensity *AI*_*in*_ (*i≠n*) for the transformation of land class *i* at the beginning of the period into a particular land class n at the end of the period within the time interval [*Y*_*t*_,*Y*_*t+1*_]; and Eq (5) calculates the average absolute transfer intensity *MAI*_*n*_ for the transformation of all other land classes, except n, into a land class n within the same time interval.
$$AI_{in}=\frac{C_{in}/(Y_{t+1}-Y_{t})}{\sum_{i=1}^{I}C_{in}}$$ (4)
$$MAI_{n}=\frac{\left[(\sum_{i=1}^{I}C_{in}-C_{nn})/(I-1)]/(Y_{t+1}-Y_{t}\right)}{\sum_{i=1}^{I}C_{in}}$$ (5)
  Where *i* represents the land class code at the beginning of the period; *n* represents the code of the transferred land class; *C*_*in*_ represents the area of land transferred from land class *i* to land class n during the time interval [*Y*_*t*_,*Y*_*t+1*_]; *C*_*nn*_ represents the area of land in land class n that has not changed during the time interval [*Y*_*t*_,*Y*_*t+1*_]; *t* represents the code of the time node at the beginning of the period; *Y*_*t*_ represents the year corresponding to the time node *t* at the beginning of the period; *Y*_*t+1*_ represents the year corresponding to the time node at the end of the period; *I* represents the number of land classes at the beginning of the period.
  Eq (6) calculates the absolute transfer intensity *AO*_*mj*_(*m≠j*) of a land class *m* at the beginning of the period into a land class j at the end of the period within the time interval [*Y*_*t*_,*Y*_*t+1*_]; and Eq (7) calculates the average absolute transfer intensity *MAO*_*m*_ of the conversion of a land class *m* into a land class other than *m* within the time interval [*Y*_*t*_,*Y*_*t+1*_].
$$AO_{mj}=\frac{C_{mj}/(Y_{t+1}-Y_{t})}{\sum_{j=1}^{J}C_{mj}}$$ (6)
$$MAO_{m}=\frac{\left[(\sum_{j=1}^{J}C_{mj}-C_{mm})/(J-1)]/(Y_{t+1}-Y_{t}\right)}{\sum_{j=1}^{J}C_{mj}}$$ (7)
  Where *m* represents the code of the transferred land class; *j* represents the code of the land class at the end of the period; *C*_*mj*_ represents the area of land transferred from land class m to land class *j* during the time interval [*Y*_*t*_,*Y*_*t+1*_]; *C*_*mm*_ represents the area of land that has not changed from land class m during the time interval [*Y*_*t*_,*Y*_*t+1*_]; and *J* represents the number of land classes at the end of the period.
2. *Relative intensity of land use/cover change*. Eq (8) calculates the relative transfer intensity *RI*_*in*_(*i≠n*) for the transformation of land class *i* at the beginning of the period into a particular land class *n* at the end of the period within the time interval [*Y*_*t*_,*Y*_*t+1*_]; and Eq (9) calculates the average relative transfer intensity *MRI*_*n*_ for the transformation of all other land classes, except *n*, into land class *n* within the same time interval.
$$RI_{in}=\frac{C_{in}/(Y_{t+1}-Y_{t})}{\sum_{j=1}^{J}C_{ij}}$$ (8)
$$MRI_{n}=\frac{\left(\sum_{i=1}^{I}C_{in}-C_{nn})/(Y_{t+1}-Y_{t}\right)}{\sum_{j=1}^{J}\left(\sum_{i=1}^{I}C_{ij}-C_{nj}\right)}$$ (9)
  Where *C*_*ij*_ represents the area of land transferred from land class *i* at the beginning of the period to land class *j* at the end of the period during the time interval [*Y*_*t*_,*Y*_*t+1*_]; *C*_*nj*_ represents the area of land transformed from land class *n* to land class *j* at the end of the period.
  Eq (10) calculates the relative transfer intensity *RO*_*mj*_(*m≠j*) for the transformation of a land class *m* at the beginning of the period into a land class *j* at the end of the period within the time interval [*Y*_*t*_,*Y*_*t+1*_]; and Eq (11) calculates the average relative transfer intensity *MRO*_*m*_ for the transformation of a land class *m* into a land class other than *m* within the time interval [*Y*_*t*_,*Y*_*t+1*_].
$$RO_{mj}=\frac{C_{mj}/(Y_{t+1}-Y_{t})}{\sum_{i=1}^{I}C_{ij}}$$ (10)
$$MRI=\frac{\left(\sum_{j=1}^{J}C_{mj}-C_{mm})/(Y_{t+1}-Y_{t}\right)}{\sum_{i=1}^{I}\left(\sum_{j=1}^{J}C_{ij}-C_{im}\right)}$$ (11)
  Where *C*_*im*_ represents the area of land transformed from land class *i* to land class *m* at the beginning of the period.
3. *Propensity and suppression of land use/cover change*. In terms of the absolute intensity of land use/cover change, from the perspective of land class *n* acquiring transfers, if land class n uniformly acquires the same area of transfers from other land classes, then for any beginning-of-period land class (*ii≠n*), the absolute intensity of transfers *AI*_*in*_ of land class n should be equal to its average absolute intensity of transfers *MAI*_*n*_, i.e., *AI*_*in*_ = *MAI*_*n*_, which indicates that the acquisition of transfers by land class *n* from each land class is equal and less affected by the land use/cover structure of the study area. If *AI*_*in*_>*MAI*_*n*_, it means that the area of land class *n* receiving transfers from land class *i* is higher than average, and it tends to receive transfers from land class *i* at the beginning of the period. Conversely, it represents that the area of land class *n* receiving transfers from land class *i* is lower than average, and its acquisition of transfers from the beginning of the period land class *i* is inhibited.
  From the point of view of the transfer out of land class *m*, if land class *m* is evenly transferred out to each end-of-period land class (*jm≠j*), the absolute transfer intensity of land class *m* should be equal to its average absolute transfer intensity, i.e., *AO*_*mj*_ = *MAO*_*m*_, indicating that land class *m* is evenly transferred out to each end-of-period land class *j*, which is less affected by the land use/cover structure of the study area. If *AO*_*mj*_>*MAO*_*m*_, it indicates that land class *m* tends to be transferred out to land class *j*, and vice versa. It indicates that its transfer out to land class *j* is inhibited.
  In terms of the relative intensity of land use/cover change, from the perspective of land class *n* acquiring transfers, if land class *n* acquires transfers from *i* based on the proportion of the area of the beginning of the period of each land class (*ii≠n*), the relative intensity of land class *n* acquiring transfers from the beginning of the period of land class *i* should be consistent with the average relative intensity of transfers of land class *n*, i.e., *RI*_*in*_ = *MRI*_*n*_, which indicates that the acquisition of transfers by land class *n* from each land class is relatively uniform, implying that the process of land class *n* acquisition transfer has an equal impact on the proportion of the area of different early-period land classes (*i* in addition to *n*). If *RI*_*in*_>*MRI*_*n*_, it indicates that land class *n* has a relative tendency to acquire transfers from beginning-of-period land class *i*, and the transformation process has a relatively large impact on the beginning-of-period area proportion of land class *i*. Otherwise, it indicates that the process of land class *n* acquiring transfers from beginning-of-period land class *i* is relatively inhibited, and the impact on the beginning-of-period area proportion of land class *i* is relatively small.
  From the point of view of the transfer out of land class *m*, if land class *m* is transferred out to land class j based on the end-of-period area proportions of each land class (*jj≠m*), the intensity of the transfer out of land class *m* to end-of-period land class *j*, *RO*_*mj*_, should be consistent with the average relative intensity of the transfer out of land class *m*, *MRO*_*m*_, that is, *RO*_*mj*_ = *MRO*_*m*_, which suggests that the process of the transfer out of land class *m* to *j* is relatively homogeneous and the process of transferring out of land class *m* to land class *j* is relatively homogeneous to the end of different end-of-period land classes (*j* except for *m*) are equally affected in terms of area proportions. If *RO*_*mj*_>*MRO*_*m*_, it indicates that land class m is relatively inclined to transfer out to *j*, and this process has a relatively large impact on the end-of-period area proportion of land class *j*. Otherwise, it indicates that the process of transferring out of land class *m* to *j* is relatively suppressed, and the impact on the end-of-period area proportion of land class *j* is relatively small.
4. *Mapping the intensity of land use/cover change*. The intensity analysis framework constructed in this paper is a deep mining and re-analyzing of the transfer matrix information, which can provide more decision-making information for land managers. However, when the analysis of LUCC in the study area involves more time nodes and land classes, it is often difficult for decision-makers to intuitively identify key and interesting spatial and temporal patterns from the complex and diverse types of changes, so it is necessary to visualize and express the computational results of the intensity analysis framework in an intuitive way, and for this reason, this paper constructs the intensity map of land use/cover change to show the key regional LUCC patterns of regional LUCC.

**Fig 4:**
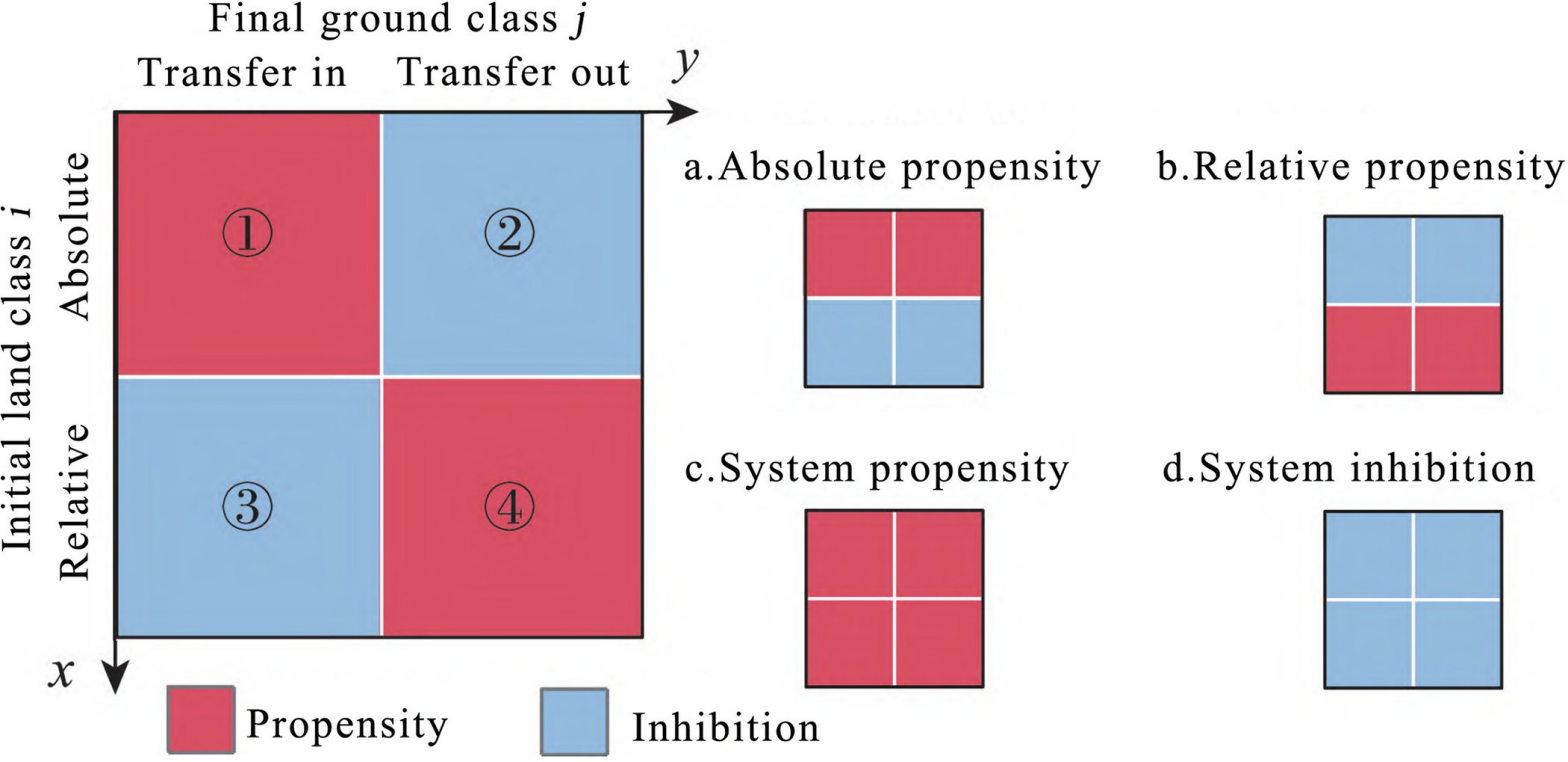
Land use/cover change intensity mapping unit.

Fig 4 shows the land use/cover change intensity mapping cells constructed according to the mutual transformation of the beginning land class *i* and the end land class *j* in a certain time interval, where the x-axis is used to identify the beginning land class and the y-axis is used to identify the end land class. Each cell of the intensity map contains four elements. Elements ① and ② are used to identify the absolute in-transition intensity and absolute out-transition intensity, and elements ③ and ④ are used to identify the relative in-transition intensity and relative out-transition intensity, respectively. The filling rules are as follows: red color represents a tendency, and blue color represents an inhibition. In particular, if elements ① and ② of the mapping unit are filled in red, it means that the absolute transfer intensity and absolute transfer intensity in the transformation process from the beginning of the period of land class *i* to the end of the period of land class *j* have tendency, and the overall transformation is characterized by absolute tendency (Fig 4A), and if elements ③ and ④ are filled in red, it means that the transformation process has relative tendency (Fig 4B). If all four elements are filled in red or blue, it represents a systematic tendency (Fig 4C) or systematic inhibition (Fig 4D).

#### 3.3.3 Spatial distribution patterns

*(1) Intensity of land expansion for construction*. The intensity index of urban built-up land expansion indicates the rate of change in the phase of the urban land area of a spatial unit, which can visualize the magnitude and speed of change in the urban land area of each spatial unit, Applicable to the comparison of temporally and spatially differentiated features of spatial units [40, 44–46]. The formula is:

$$U_{i}=\frac{DU_{ea}\times 100}{d_{t}\times L_{ta}}$$ (12)

Where *U*_*i*_ is the intensity of urban construction land expansion; *DU*_*ea*_ is the area of construction land expansion in a certain time period; *d*_*t*_ is the time period (generally in years); *L*_*ta*_ is the total land area of the study unit.

*(2) LISA time path*. *LISA* (local spatial autocorrelation) time path is a continuous representation of the positional shift of a spatial unit in *Moran’s I* scatter plot. By visualizing the pairwise movement of an attribute value of a spatial unit and its spatial lag, the strength and direction of spatial-temporal interaction between neighboring domains can be revealed, thus making the traditional static *LISA* more dynamic. *LISA* time path indexes include the relative length, curvature, and direction of the moving path. The length of the *LISA* time path reflects the dynamic characteristics of the local spatial structure of the city, the curvature reflects the fluctuating characteristics of the local spatial structure, and the direction of the leap reflects the dynamical characteristics of the urban local spatial structure. The *LISA* time path length can reflect the dynamic characteristics of the local spatial structure of the city, the curvature reflects the fluctuation characteristics of the local spatial structure of the city, and the direction of the jump reflects the integration characteristics of the evolution of the local spatial structure of the city [47], and the expressions are as follows:

$$d_{i}=\frac{N\sum_{t=1}^{T-1}d(L_{i,t},L_{i,t+1})}{\sum_{i=1}^{N-1}\sum_{t=i}^{T-1}d(L_{i,t},L_{i,t+1})}$$ (13)

$$\varepsilon_{i}=\frac{\sum_{t=1}^{T-1}d(L_{i,t},L_{i,t+1})}{d(L_{i,t},L_{i,T})}$$ (14)

Where *d*_*i*_ and *ε*_*i*_ are the path length and curvature of city *i*, respectively; *N* is the number of study units, *N* = 114 in the text; *T* is the length of the study time; *L*_*i*_, _*t*_ are the *LISA* coordinates of city *i* at time *t*; *d(L*_*i*_,_*t*_, *L*_*i*_,_*t+1*_*)* is the moving distance of city *i* from time *t* to *t+1*. If the moving length of prefecture-level city *i* exceeds the mean value of all cities during the study period, *di*>1, and vice versa *di*<1; longer relative lengths indicate more dynamic local spatial dependencies and spatial structures. If the moving path of city i is non-linear, *ε*_*i*_>1 and vice versa *ε*_*i*_<1; larger values of *ε*_*i*_ indicate a more dynamic local spatial dependency direction and a more volatile growth process.

*(3) LISA time leap*. Based on *LISA*, *Rey* embedded the attributes such as distance, direction, and cohesion of each spatial unit in the *Moran’s I* scatterplot within a specific time interval into a traditional Markov chain and proposed local Markov transfer and spatio-temporal leapfrogging, which are used as a decomposition method to reveal the spatial dependence of geographic phenomena [48]. *Rey* classified the spatio-temporal jump cases into *Type*_*0*_, *Type*_*1*_, *Type*_*2*_, and *Type*_*3*_. *Type*_*0*_ indicates that the city itself and its neighbors do not undergo inter-morphological jumps over time, and all of them are located in the main diagonal of the transfer matrix; *Type*_*1*_ indicates that the city itself jumps, but its neighbors remain unchanged, including HH_t_→LH_t+1_,HL_t_→LL_t+1_,LH_t_→HH_t+1_,LL_t_→HL_t+1_; *Type*_*2*_ indicates that the city itself is unchanged but the neighborhood jumps, including HH_t_→HL_t+1_,HL_t_→HH_t+1_,LH_t_→LL_t+1_,LL_t_→LH_t+1_; *Type*_*3*_ indicates that the city itself and the neighborhood both jump, and this type can be further divided into *Type*_*3A*_ and *Type*_*3B*_ types, the former indicating that the city itself and the neighboring region in the same direction, including HH_t_→LL_t+1_、LL_t_→HH_t+1_; the latter indicates that the two jump in the opposite direction, including HL_t_→LH_t+1_、LH_t_→HL_t+1_. *Rey* defines spatio-temporal flow (*SF*) and cohesion (*SC*) in the regional system to indicate the spatial pattern of the study object’s path-dependence and locking characteristics, and the number of type of leaps within the study time period to the total number of possible leaps (*m*) in the system [47].

$$SF=\frac{Type_{1}+Type_{2}}{m}$$ (15)

$$SC=\frac{Type_{0}+Type_{3A}}{m}$$ (16)

where *Type*_*0*_, *Type*_*1*_, *Type*_*2*,_ and *Type*_*3A*_ denote the number of leaps, respectively.

#### 3.3.4 Research on the driving forces of land use change based on PLUS model

The PLUS model, developed by the HMSCIL @CUG Laboratory at China University of Geosciences, is a land use simulation model capable of uncovering the drivers behind various types of land use changes and simulating changes at the level of land use patches. This model comprises two major modules: the Land Expansion Analysis Strategy (LEAS) and the Cellular Automata Random Seed-based (CARS) model, which works in tandem to analyze land use expansion and simulate changes across various types of land use patches. The LEAS module, adept at extracting land expansion dynamics between two periods and conducting sampling, employs the Random Forest algorithm to unearth and acquire development probabilities for various land uses, along with the contribution rates of driving factors. The CARS module, integrating random seed generation and a threshold reduction mechanism, simulates the autonomous generation of patches under the constraint of development probabilities [49].


$$W_{i}=\frac{TA_{i}-TA_{\min}}{TA_{\max}-TA_{\min}}$$ (17)

In the equation, *W*_*i*_ represents the domain weight for the *i*-th land type. *T*A_i_ stands for the expansion area of the *i*-th land use category. *TA*_*min*_ corresponds to the minimum expansion area for each land use category. *TA*_*ma*x_ corresponds to the maximum expansion area for each land use category.

## 4 Results

### 4.1 The slope spectrum characteristics of construction land in Chongqing’s central urban area

**Fig 5:**
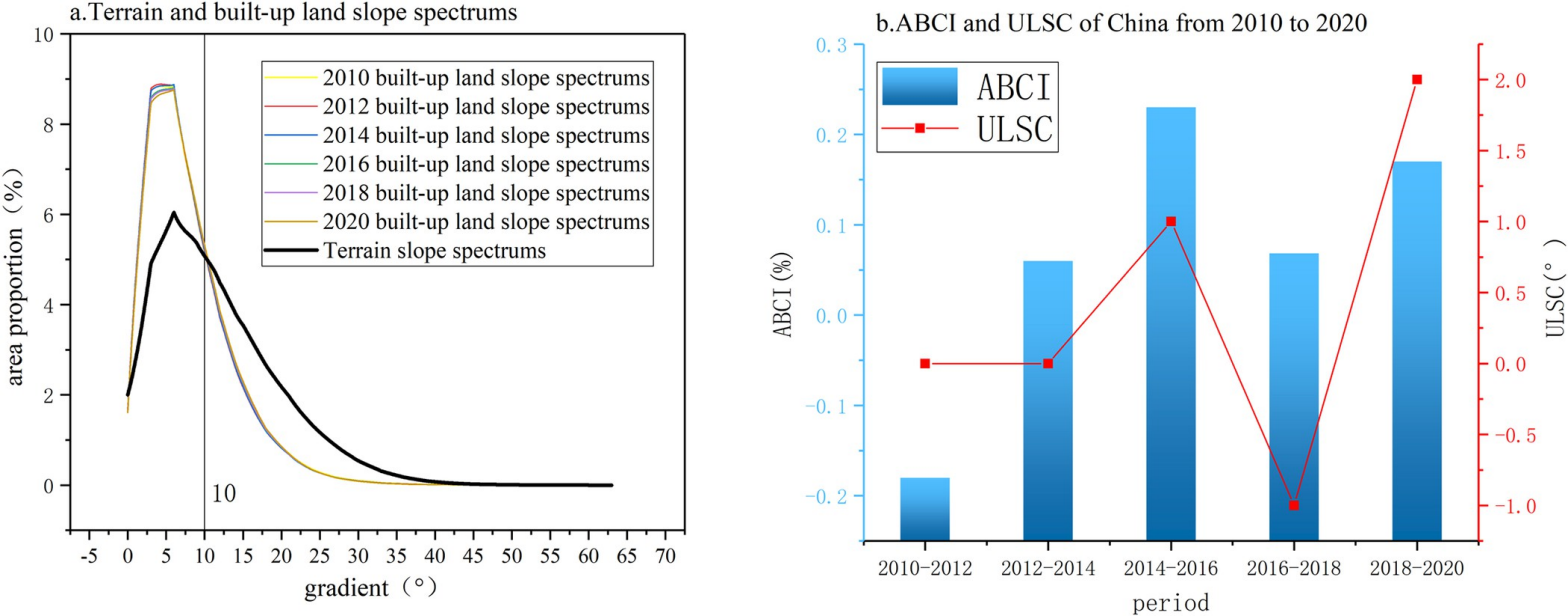
Terrain and built-up land slope spectrums, BCI and ULSC from 2010 to 2020.

In the period from 2010 to 2020, the distribution pattern of the developed land slope spectrum in the central urban area of Chongqing is illustrated in Fig 5A. According to the statistics, the average slope of developed land distribution in the central urban area of Chongqing was 7.93° in 2010 and increased to 8.00° in 2020. This indicates a rise of 0.07° in average slope over the span of ten years. The topographic and developed land slope spectrum curves exhibit skewed distributions, with their peaks falling within the 7°-8° slope range. Beyond 8°, as the slope increases, the proportion of developed land gradually decreases, eventually approaching zero. The intersection point of the topographic slope spectrum and the developed land slope spectrum occurs at around 10°, indicating that most developed land in the central urban area of Chongqing is distributed on slopes below 10°. Specifically, the proportion of developed land with slopes below 10° was 74.05% in 2010, 74.41% in 2012, 74.28% in 2014, 73.82% in 2016, 73.69% in 2018, and 73.34% in 2020, relative to the total area. From 2010 to 2020, the proportion of developed land with slopes above 10° exhibited fluctuations over time. Specifically, there was an increase of 0.36% from 2010 to 2012, a 0.13% increase from 2012 to 2014, a 0.46% increase from 2014 to 2016, a 0.13% increase from 2016 to 2018, and a 0.35% increase from 2018 to 2020. An analysis of the changes in the upper slope limit of developed land and the Developed Land Climbing Index (Fig 5B) in the central urban area of Chongqing for different periods reveals that from 2010 to 2012, the Developed Land Climbing Index was -0.33%, and the change in the upper slope limit was 0°. From 2012 to 2014, the Developed Land Climbing Index was 0.19%, with a change in the upper slope limit of 0°. For 2014–2016, the Climbing Index was 0.56%, with a 1° change in the upper slope limit. From 2016 to 2018, the Climbing Index was 0.17%, accompanied by a -1° change in the upper slope limit. Finally, in 2018–2020, the Climbing Index was 0.42%, with a change in the upper slope limit of 2°. The results indicate that in the central urban area of Chongqing, developed land gradually expanded towards lower slope areas from 2010 to 2012 and shifted towards higher slope areas from 2012 to 2020. Moreover, the intensity of climbing for developed land significantly increased after 2012, reaching its peak from 2014 to 2016.

### 4.2 LUCC intensity mapping of Chongqing downtown 2010–2020

**Fig 6:**
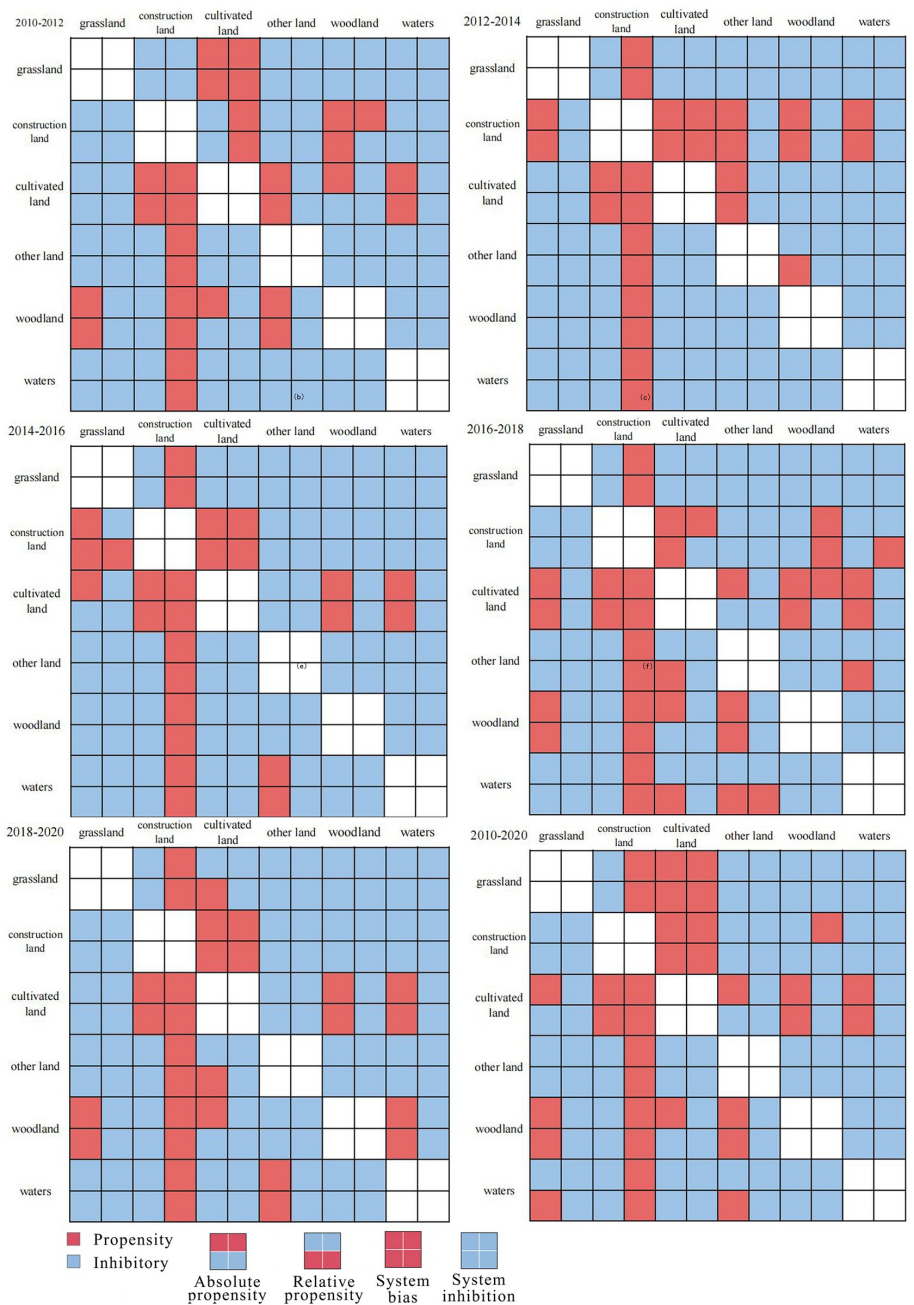
Mapping the intensity of land use/cover change in the central urban area of Chongqing, 2010–2020.

Firstly, to calculate the land use/cover change transition matrices for the central urban area of Chongqing from 2010–2012, 2012–2014, 2014–2016, 2016–2018, 2018–2020, and the overall period of 2010–2020, we need to analyze the transitions between different land use/cover categories during these time intervals.Harnessing the capabilities of the Intensity Map software, we embark on an illuminating journey to compute the land use/cover change intensity maps for Chongqing’s central urban district. These temporal snapshots, spanning from 2010–2012, 2012–2014, 2014–2016, 2016–2018, and 2018–2020, will unravel the dynamic interplay of transformations. Ultimately, these intensity maps will be woven together to form a comprehensive tapestry, encapsulating the intricacies of land evolution over the entire decade at the heart of Chongqing. Indeed, as illustrated in Fig 6A–6F, the transformation characteristics among various land classes within the maps exhibit distinct heterogeneity. Given the focus of this study on urban expansion, our analysis will be honed exclusively on the transformation characteristics of construction land. As depicted in Fig 6F, for 2010–2020, a systematic propensity for the transformation of cropland into construction land emerged. Additionally, grassland to construction land, woodland to construction land, water bodies to construction land, and other land types to construction land exhibit both absolute and relative tendencies towards outward transformations. In a parallel fashion, the transformation of construction land into cropland demonstrates a systematic inclination. Conversely, the conversions from construction land to grassland, woodland, water bodies, and other land types display a systematic restraint delineated by the distinctive transformation characteristics.

### 4.3 Spatial distribution patterns

#### 4.3.1 Trend analysis

**Fig 7:**
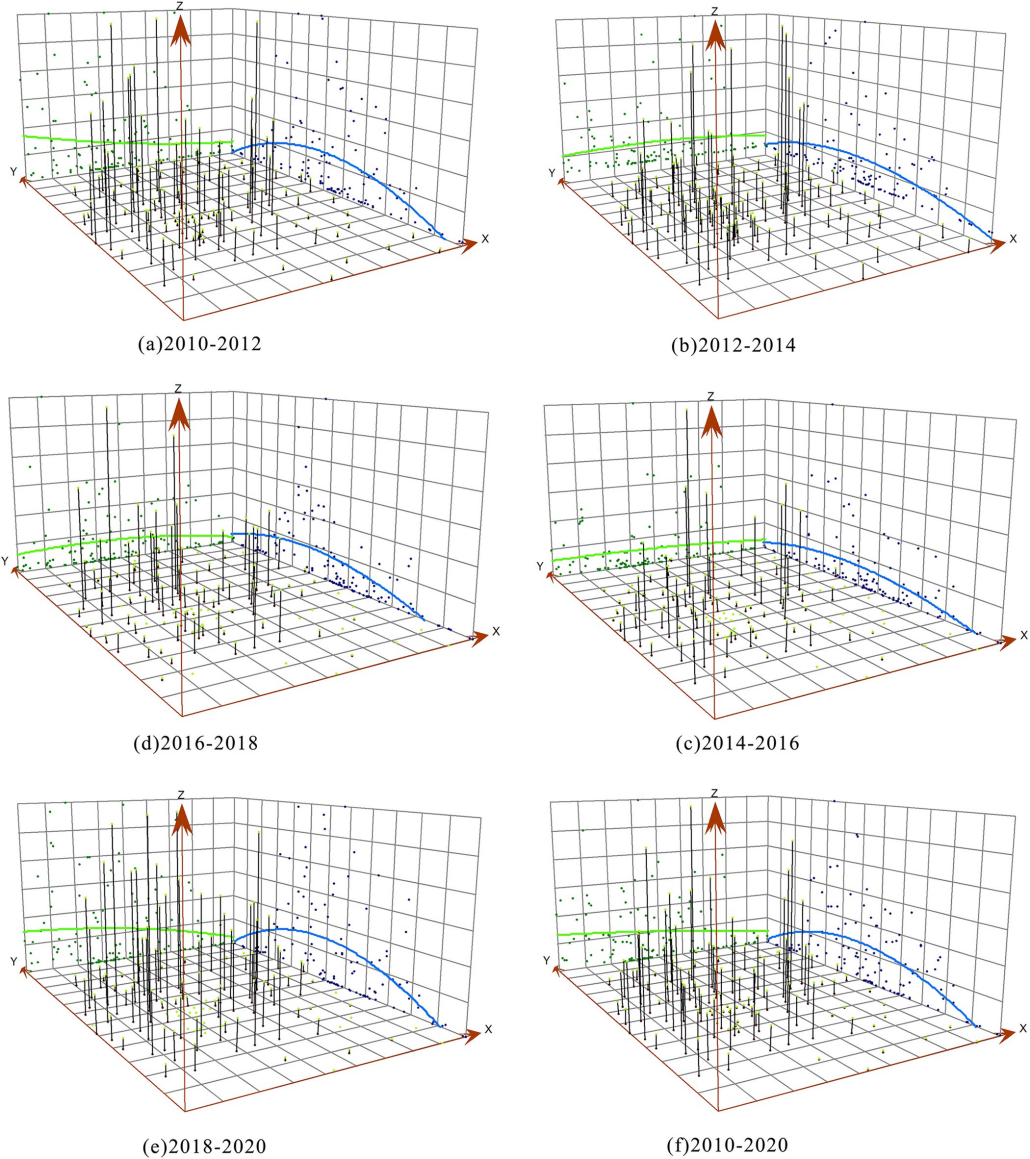
Trend surface analysis of the expansion intensity of construction land in the center of Chongqing city.

Certainly, we will apply Eq (12) to calculate the intensity of construction land expansion for the study area during the following time intervals: 2010–2012, 2012–2014, 2014–2016, 2016–2018, 2018–2020, and the entire decade from 2010 to 2020. This rigorous analysis will provide a comprehensive understanding of the dynamics of construction land expansion across these temporal frames. Subsequently, we will harness the capabilities of ArcGIS 10.8, employing its trend analysis functionality to generate the trend analysis maps as illustrated in Fig 7A–7F. These maps will illuminate the evolving trends in construction land expansion across the specified time periods, visually representing the study’s findings. Overall, the intensity of construction land expansion (Z-axis) within the central urban area of Chongqing exhibits a decreasing trend from west to east (X-axis) over time. This pattern is consistent, except from 2012 to 2014, during which the intensity initially increased from west to east before showing a subsequent decline. Particularly noteworthy is the distinct reduction in the index in the eastern regions of the study area. In the north-south direction (Y-axis) within the study area, there is a gradual transition from higher values in the northern parts to higher values in the southern regions, ultimately forming a "U-shaped" curve with a decreasing trend from the center towards the periphery. The pronounced variations in different directions on the trend surfaces reflect the increasing imbalance in the intensity of construction land expansion within the central urban area of Chongqing. This emphasizes the significant presence of spatial heterogeneity in the expansion patterns, underscoring the complexity and diversity of land dynamics in the region.

#### 4.3.2 Analysis of spatial distribution patterns

**Fig 8:**
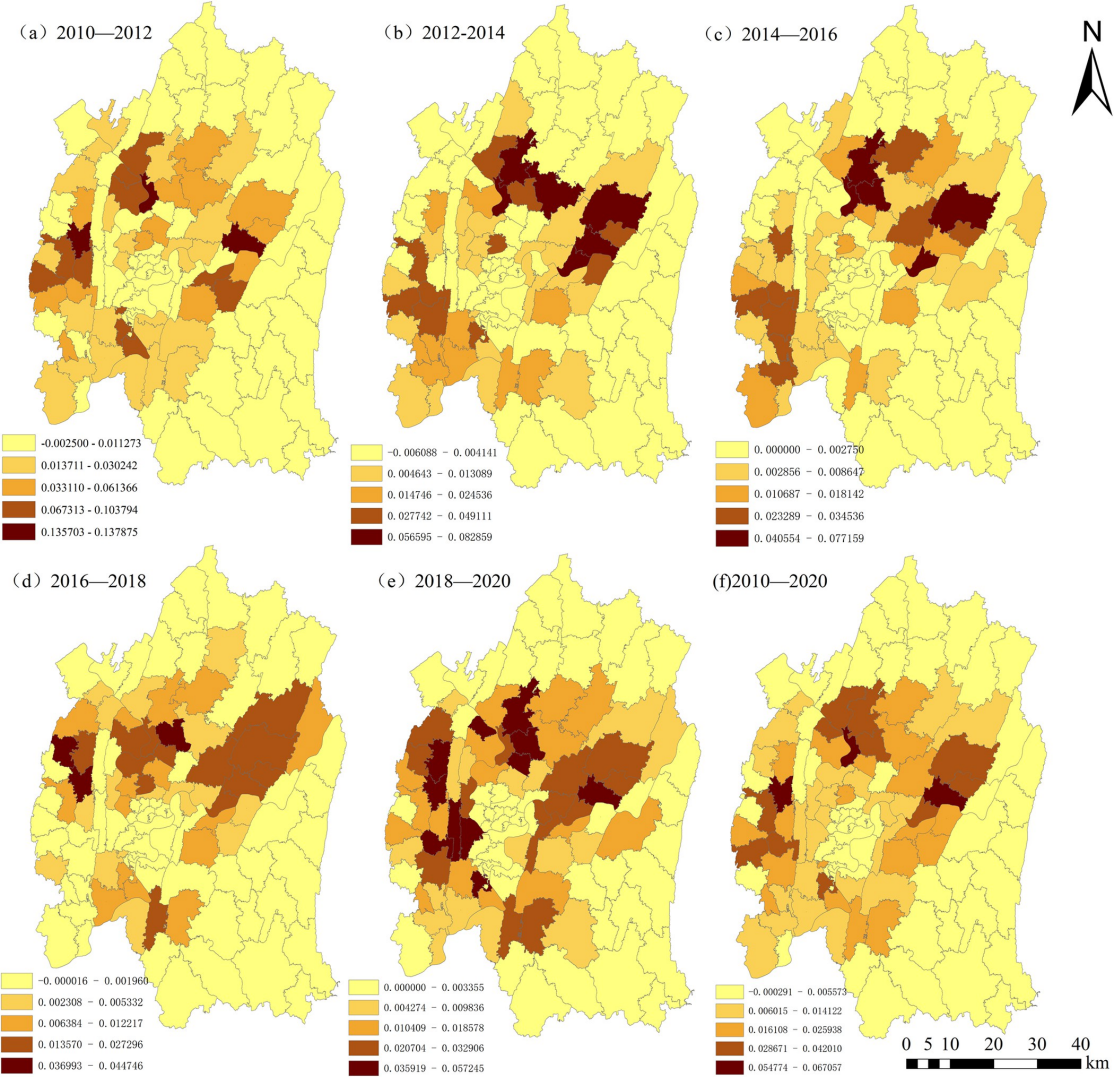
Spatial differentiation pattern of expansion intensity of construction land in the central urban area of Chongqing (2010–2020). Note: All administrative boundary data comes from Resource and Environment Science and Data Center (https://www.resdc.cn/), and the map boundary has not been changed.Cartographic software:ArcGIS.

As depicted in Fig 8, from 2010 to 2020, the intensity of construction land expansion within the central urban area of Chongqing exhibits a continuous upward trend. It evolves from a low-speed growth to a gradually accelerating pace, demonstrating a discernible spatial clustering effect and alternation in distribution patterns. From 2010 to 2012, high-intensity expansion zones were primarily concentrated in Jiangbei District, Yubei District, and Shapingba District. In a manner similar to 2012–2014, during 2014–2016, the high-intensity expansion zones were predominantly concentrated in the eastern part of Jiangbei District and the southern part of Yubei District. In 2016–2018, the high-intensity expansion zones shifted towards the southern part of Beibei District and the western part of Shapingba District. During 2018–2020, the high-intensity expansion zones diffused outward in a concentric manner, with the primary concentration still observed in Yubei District, Jiangbei District, and Shapingba District. From 2010 to 2020, areas with low-intensity expansion were primarily concentrated in the northern part of Yubei District, the northern part of Beibei District, the southern part of Jiulongpo District, the eastern part of Nan’an District, and much of the area in Banan District. On an overarching scale, over the course of 10 years, the disparity between high-intensity and low-intensity expansion zones within the central urban area of Chongqing has notably widened. The trajectory has evolved from a relatively balanced, low-speed expansion during 2010–2012 to a rapid, uneven expansion from 2014 to 2020. The high-intensity zones progressively clustered towards the eastern part of Jiangbei District, the southern part of Yubei District, and Shapingba District. Conversely, the low-intensity zones predominantly occupied the Beibei District and Banan District.

### 4.4 Spatial correlation and dynamic characterization

#### 4.4.1 Spatial autocorrelation analysis

*(1) Global spatial autocorrelation*. As indicated in Table 3, the global Moran’s I index for the urban expansion intensity index from 2010 to 2020 consistently exhibits positive values, which are significant at the 5% level. This signifies the presence of a significant positive spatial correlation and spatial clustering patterns within the data. The gradual increase in the global Moran’s I index suggests a rising trend in the spatial clustering of construction land expansion intensity within the central urban area of Chongqing. This trend indicates a growing concentration and notable spatial clustering pattern in the intensity of construction land expansion.

**Table 3:** Global Moran’s I index of construction land expansion intensity in the center of Chongqing city.

Time	Moran’s I	Z	P
2010–2012	0.207800	4.618066	0.000004
2012–2014	0.200239	4.495641	0.000007
2014–2016	0.247247	5.608590	0.000000
2016–2018	0.259115	5.835297	0.000000
2018–2020	0.289985	6.324405	0.000000
2010–2020	0.347719	7.593652	0.000000

**Fig 9:**
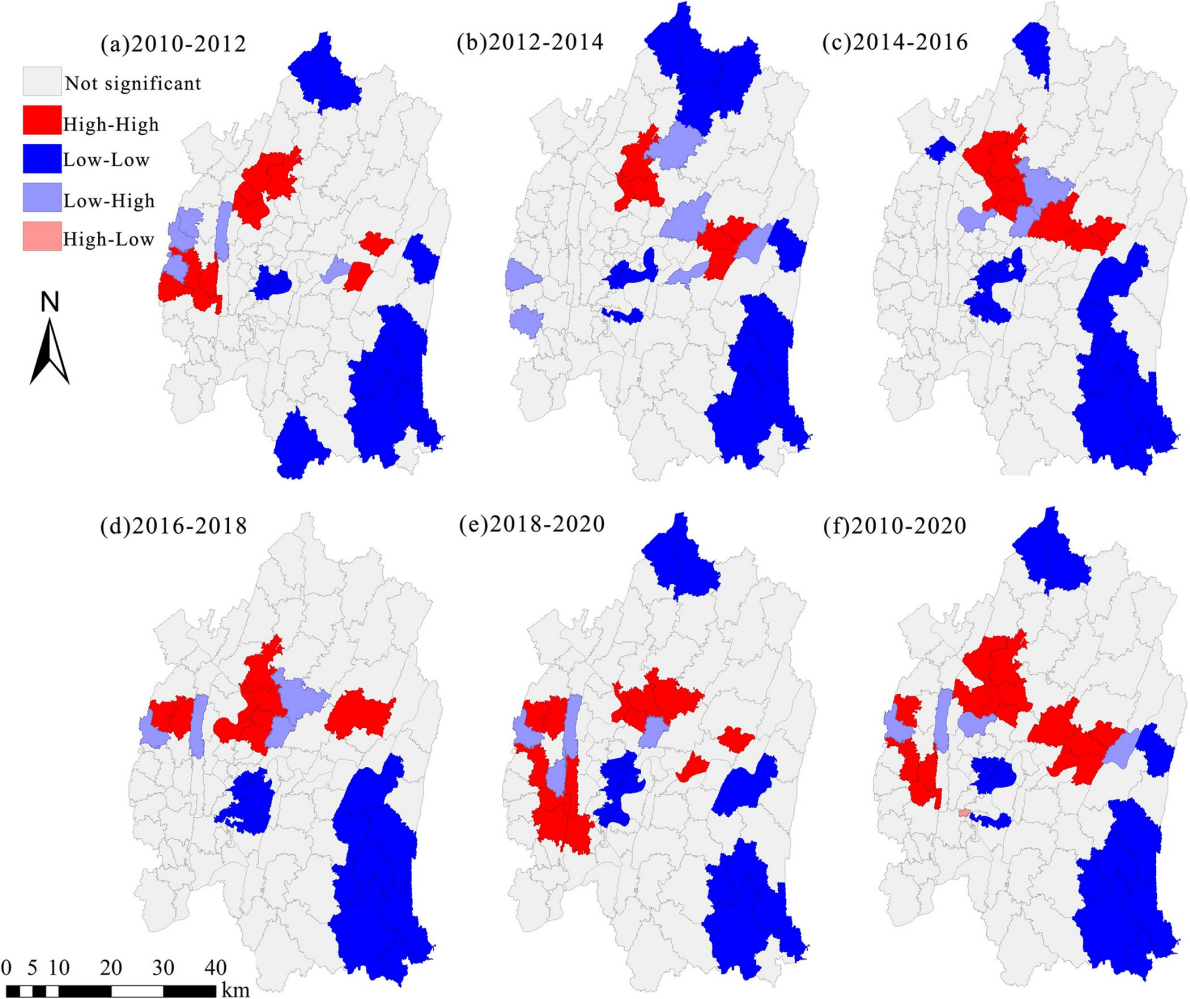
Characteristics of local spatial correlation of construction land expansion intensity in central Chongqing (2010–2020). Note: All administrative boundary data comes from Resource and Environment Science and Data Center (https://www.resdc.cn/), and the map boundary has not been changed.Cartographic software:ArcGIS.

*(2) Local spatial autocorrelation*. Utilizing local spatial autocorrelation analysis, we have generated Local Indicators of Spatial Association (LISA) cluster maps to investigate the localized spatial clustering characteristics of construction land expansion intensity within the central urban area of Chongqing. These LISA cluster maps will provide valuable insights into the specific regions exhibiting significant spatial clustering patterns. As depicted in Fig 9A–9F, the local spatial clustering characteristics of construction land expansion intensity are notably evident. Generally, these patterns are dominated by high-high and low-low clusters. From 2010 to 2020, areas exhibiting high-high clustering, with Yuzhong District at the center, gradually shifted in a clockwise direction from Shapingba District towards Yubei District and Jiangbei District. Regions characterized by low-low clustering are predominantly concentrated in the northern parts of Banan District and Yubei District. There are scattered occurrences in the Shapingba District, with a relatively dispersed distribution. Overall, there is a notable spatial correlation in the intensity of construction land expansion within the central urban area of Chongqing. This level of correlation allows for a more in-depth analysis of the contribution and spatial structure of each unit. Subsequently, in the following sections, we will delve into the dynamic, dependent, and spatially integrated characteristics of the local spatial structure through LISA time paths and spatiotemporal transitions.

#### 4.4.2 LISA time path and time leap analysis

*(1) Spatial characterization of LISA time paths*. The relative length of LISA time paths reflects the dynamic characteristics of the local spatial structure of construction land expansion intensity in each township. Fig 7A and 7B shows that the relative lengths are categorized into four classes using the natural breaks method. During the study period, there were 47 townships within the central urban area of Chongqing where the relative lengths were above the mean value, accounting for 41.22% of the total. In terms of spatial distribution, these townships with above-average relative lengths are primarily located in the northern part of Nan’an District, the southern part of Yubei District, the southern part of Beibei District, the eastern part of Jiangbei District, the western part of Shapingba District, as well as in Dadukou District, the western part of Jiulongpo District. There are sporadic occurrences in Banan District, indicating a relatively dynamic local spatial structure. There are 67 townships with relative lengths below the mean value, constituting 58.78% of the total. These townships are mainly distributed across the majority of Banan District, the northern part of Yubei District, the northern part of Beibei District, and the established urban areas within the central urban area of Chongqing. They exhibit a relatively stable local spatial structure. This result aligns with the overall spatial distribution characteristics of construction land expansion intensity within the urban area, demonstrating consistency in the local spatial structure dynamics. Looking at the average values of the nine districts within the central urban area of Chongqing, the order is as follows: Shapingba District (1.8526) > Jiulongpo District (1.3251) > Yubei District (1.3251) > Nan’an District (0.9534) > Beibei District (0.9193) > Jiangbei District (0.9073) > Dadukou District (0.7555) > Banan District (0.357) > Yuzhong District (0.1734). This indicates that Shapingba District, Jiulongpo District, and Yubei District exhibit a strong level of instability in their local spatial structures, while Nan’an District, Beibei District, Jiangbei District, and Dadukou District follow, and Banan District and Yuzhong District have relatively stable local spatial structures. Overall, among the 114 townships within the central urban area of Chongqing, the cities with higher levels of economic development exhibit a notable degree of dynamism and instability in their local spatial structures. Conversely, townships with lower levels of economic development tend to possess a relatively stable local spatial structure. Additionally, due to the completion of urbanization in Yuzhong District before 2010, this economically developed district also exhibits a relatively stable local spatial structure.

**Fig 10:**
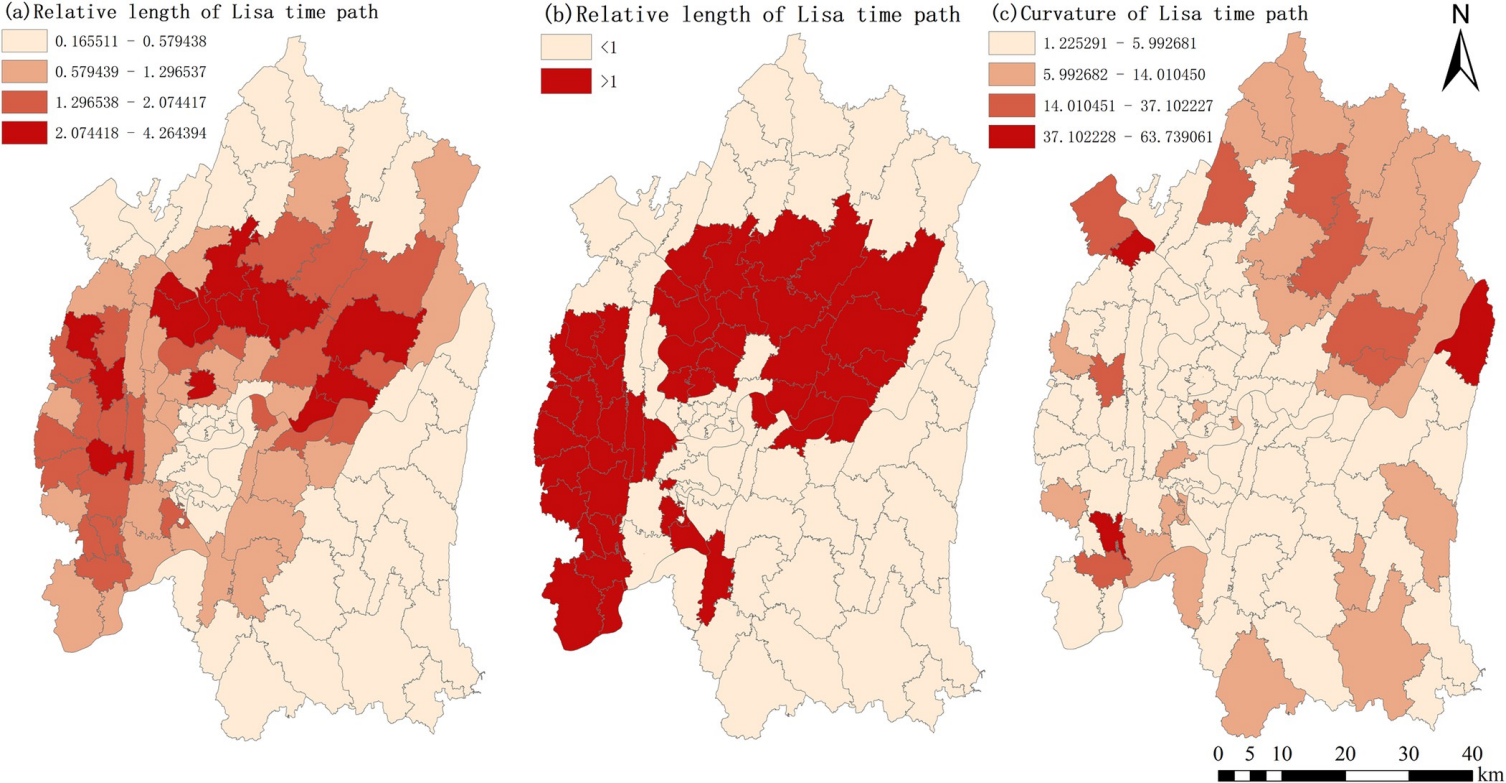
Relative length and curvature of Lisa time path. Note: All administrative boundary data comes from Resource and Environment Science and Data Center (https://www.resdc.cn/), and the map boundary has not been changed.Cartographic software:ArcGIS.

Using the natural breaks method in ArcGIS, the curvature of construction land expansion intensity for the 114 townships within the central urban area of Chongqing has been categorized, as illustrated in Fig 10(C). With curvature values exceeding 1 for all townships within the central urban area of Chongqing, it is evident that construction land expansion intensity demonstrates pronounced shifting and variation characteristics across the region. The townships with higher curvature values are primarily concentrated in the northern part of the central urban area of Chongqing. There are also scattered occurrences in the western part of the central urban area. These areas are characterized by hilly terrain, which limits urban development space and contributes to significant fluctuations in spatial dependence directions. The townships surrounding the built-up areas within the central urban area of Chongqing exhibit lower curvature values, indicating relatively stable spatial dependence and directional changes. When considering specific districts, the order is as follows: Beibei District (11.2343) > Jiulongpo District (9.9803) > Yubei District (8.762) > Banan District (6.8851) > Jiangbei District (5.9269) > Dadukou District (5.0131) > Shapingba District (4.4175) > Nan’an District (3.0065) > Yuzhong District (2.1553). This implies that Beibei District, Jiulongpo District, Yubei District, Banan District, Jiangbei District, and Dadukou District exhibit fluctuating spatial dependence, with noticeable overflow effects from local structures. Nan’an District and Yuzhong District follow, with Yuzhong District displaying relatively stable spatial dependence. In general, the local spatial directional changes in construction land expansion intensity within the central urban area of Chongqing exhibit relative stability, indicating a degree of path dependence.

*(2) Spatial characterization of the LISA space-time leap*. As shown in Table 4, in 2020, the most common type of transition is Type 0, which indicates that construction land and its surroundings do not undergo morphological transitions over time. The probability of this type of transition occurring is 74%, suggesting that the construction land expansion intensity within the central urban area of Chongqing remains relatively stable. Next are Type 1 and Type 2, where either construction land or its surroundings experience transitions, with probabilities of 12% each. Type 3, where both the city and its surroundings undergo transitions, has the smallest probability, at only 2%. In the overarching context, there exists a 74% probability that the intensity of urban land expansion in Chongqing’s central city area has not undergone a significant leap. Temporally and spatially, cohesion remains as high as 74%. This signifies a relative stability in the spatial positioning of various cities within the region, demonstrating a pronounced degree of path dependency. This outcome also reveals that in the central city area of Chongqing, most urban land expansion intensities have failed to exhibit significant temporal and spatial leaps. The expansion intensity of construction land remains relatively stable in terms of the local spatial patterns of neighboring cities. Various townships find it challenging to alter their inherent positions within the framework.

**Table 4:** Local Moran’s I transfer probability matrix with temporal jumps.

period	t/t+1	HL	HH	LL	LH	Type	number	proportion	SF	SC
2010–2020	HL	0.60	0.07	0.06	0.01	Type0	840	0.74	0.85	0.74
	HH	0.05	0.03	0.00	0.01	Type1	140	0.12		
	LL	0.04	0.00	0.10	0.00	Type2	140	0.12		
	LH	0.01	0.02	0.00	0.02	Type3	20	0.02		

### 4.5 Analysis of the driving factors for the expansion of urban construction land in the central urban area of Chongqing

The selection of driving factors for urban construction land expansion should not only reflect the natural geographical characteristics of the central urban area of Chongqing, but also comprehensively consider the driving effects of economic and social development conditions. Based on the actual situation of the central urban area of Chongqing, the availability of data, and the selection of driving factors in relevant research, this study selected the driving factors shown in Table 1.

**Fig 11:**
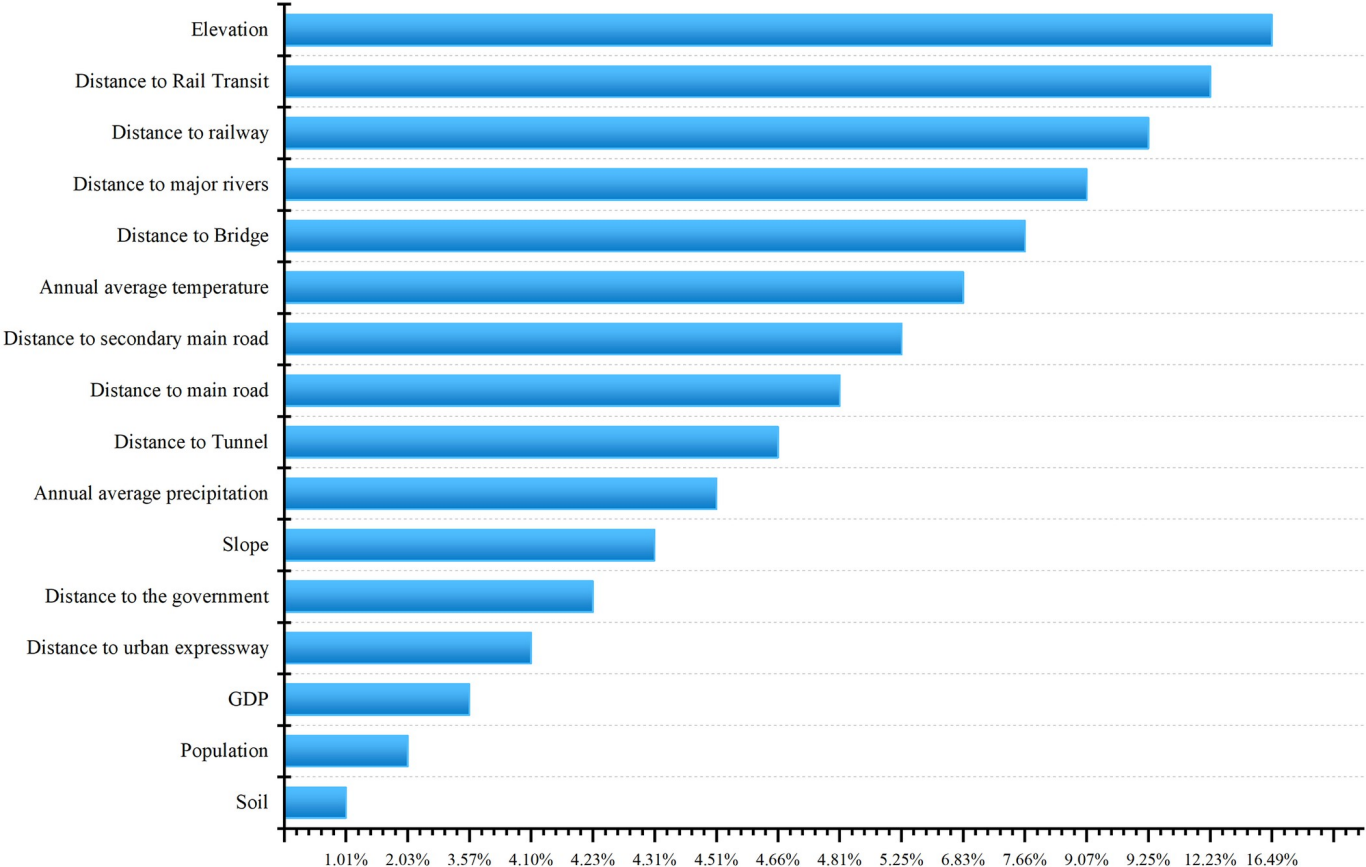
Contribution percentage of drivers of construction land area growth from 2010 to 2020.

Between 2010 and 2020, there were significant changes in the construction land area in the central city area of Chongqing. As a typical large mountainous city spanning the Yangtze River, in order to further explore the driving factors behind the expansion of construction land in this area, land use data from 2010 and 2020, along with driving force data, were input into the PLUS model. This yielded the proportion of various driving factors between 2010 and 2020. The larger the proportion of a driving factor, the greater its contribution to the expansion of construction land. Conversely, the smaller the proportion of a driving factor, the less its contribution to the expansion of construction land. The results of the driving forces behind the urban construction land changes in the central city area of Chongqing are illustrated in Fig 11. From 2010 to 2020, the main contributing factors to the expansion of construction land were elevation (16.49%), distance to rail transit (12.23%), distance to railways (9.25%), distance to major rivers (9.07%), distance to bridges (7.66%), annual average temperature (6.83%), distance to secondary roads (5.25%), and distance to main roads (4.81%). Overall, due to its status as a typical large mountainous city spanning the Yangtze River, the expansion of urban construction land in the central city area of Chongqing is significantly influenced by factors such as topography, rivers, tunnels, and rail transit. These elements play a substantial role in shaping the landscape and development of the region.

## 5 Conclusion and discussion

### 5.1 Conclusion

This study, based on annual land change survey data for the central city area of Chongqing in the years 2010, 2012, 2014, 2016, 2018, and 2020, as well as township-level administrative data and topographic data, employed GIS spatial analysis techniques. From the perspectives of vertical and horizontal expansion of construction land, it analyzed the spatial growth process and driving factors in the construction space of the central city area of Chongqing. The research findings are as follows:

1. From the perspective of vertical expansion of construction land, it is observed that in the central city area of Chongqing, from 2010 to 2012, construction land gradually extended towards lower slope areas, and from 2012 to 2020, it extended towards higher slope areas. Furthermore, the intensity of uphill construction land development significantly increased after 2012, reaching its peak between 2014 and 2016.
2. From 2010 to 2020, there was a systematic tendency to convert farmland into construction land. Similarly, grassland to construction land, woodland to construction land, water bodies to construction land, and other land types to construction land exhibited absolute and relative tendencies for land conversion. On the other hand, the conversion of construction land back into farmland displayed a systematic tendency. Additionally, the conversion of construction land to grassland, woodland, water bodies, and other land types exhibited systematic inhibitory characteristics.
3. Since 2010, the intensity of construction land expansion in the central city area of Chongqing has shown a steady growth trend, evolving from slow and balanced growth to fast and unbalanced growth, leading to a gradual widening of spatial disparities. The variations in expansion intensity across townships exhibit spatial alternations, with high-value areas gradually congregating towards the eastern part of Jiangbei District, the southern part of Yubei District, and Shapingba District. Low-value areas are concentrated in Beibei District and Banan District.
4. From the analysis of local neighborhood characteristics, it is observed that most townships are in a relatively stable spatial state, with no significant leaps occurring in the expansion intensity of construction land. This indicates the presence of a spatial lock-in feature in the expansion of construction land. The central city area of Chongqing exhibits strong overall spatial cohesion, with neighboring townships primarily characterized by positive synergistic growth. Polarization effects and spatial spillover effects in Yubei District, Nan’an District, Jiangbei District, and Shapingba District play important roles in shaping the spatial dynamics of the region.
5. This study utilized the PLUS model to analyze the urban land expansion patterns and driving forces in the central city area of Chongqing. As a typical large mountainous city spanning the Yangtze River, the expansion of urban construction land in this area is significantly influenced by factors such as elevation (16.49%), distance to rail transit (12.23%), distance to railways (9.25%), proximity to major rivers (9.07%), distance to bridges (7.66%), annual average temperature (6.83%), distance to secondary roads (5.25%), and distance to main roads (4.81%). These factors play a substantial role in shaping the region’s urban development landscape.

### 5.2 Discussion

This study, focusing on the spatial differentiation and correlation characteristics of vertical and horizontal expansion of construction land, selected township-level administrative regions within the central city area of Chongqing as research units. It employed time paths and spatiotemporal transitions to investigate the dynamics, directional dependencies, and spatial integration characteristics of local spatial structural changes in construction land expansion intensity over the past decade. This research serves as a valuable addition and enrichment to previous studies, which often emphasized overall spatial patterns while overlooking dynamic changes in local structures. In the course of this study, it was observed that since 2010, the central city area of Chongqing has witnessed a substantial increase in both the level and quality of construction land expansion. This region exhibits certain distinct features compared to other areas in the country. However, due to rapid development and unregulated sprawl, it faces challenges such as insufficient potential for future construction space and inefficiencies in spatial utilization. These issues exert significant pressure on the current resource environment, which is detrimental to the sustainable development of the economy and society in the region. With the introduction and implementation of China’s New Urbanization Policy, there is a growing emphasis on strict control of the scale of new urban construction land across the country. In the central city area of Chongqing, the expansion of construction land should now prioritize intensive and sustainable development to align with these policy goals.

In this context, policy recommendations for optimizing the development of the central urban area of Chongqing are proposed:

1. There is a need to intensify the formulation and implementation management of land and spatial planning, enhancing the sustainable development and conservation of natural resources within Chongqing’s central urban area. This approach will facilitate the seamless integration of urban and rural functional spaces, promoting a harmonious coexistence.
2. It is imperative to formulate judicious policies that curtail further development on hilly terrain, safeguarding the ecological integrity of Chongqing’s "Four Mountains." Such policies should strike a balance between urban expansion and ecological preservation, ensuring the sustainability of this unique natural landscape.
3. Expediting the development of diverse river and mountain crossing routes is essential to enhance accessibility to the Western Science City and the Liangjiang New Area, promoting economic synergies among different districts within Chongqing’s central urban area. These transportation links will facilitate collaboration and foster mutual growth.
4. The formation of urban construction land expansion intensity is a complex process driven by urbanization. The unique policy factors adopted by local governments are challenging to quantify and evaluate within this mechanism. Further in-depth research and exploration will be necessary for future studies to refine and delve into this matter.

Due to limitations in data acquisition, resources, time, and the length of this article, there are still numerous aspects of the PLUS model that require further research. The factors influencing construction land expansion are highly intricate, and this study has quantitatively analyzed only a few aspects, such as topography, social, and economic factors. The study did not clearly elucidate whether the driving force behind construction land expansion is positive or negative, and the selection of driving factors was somewhat arbitrary, necessitating further investigation. In future research, efforts will be intensified to enhance the scientific and comprehensive selection of driving factors, aiming for a more precise understanding of the Driving factors behind construction land expansion for each factor.

## Supporting information

S1 File Data set. (ZIP)

S2 File Spatial_driving_factors_part1. (ZIP)

S3 File Spatial_driving_factors_part2. (ZIP)
